# Two accurate sequence, structure, and phylogenetic template-based RNA alignment systems

**DOI:** 10.1186/1752-0509-7-S4-S13

**Published:** 2013-10-23

**Authors:** Lei Shang, David P Gardner, Weijia Xu, Jamie J Cannone, Daniel P Miranker, Stuart Ozer, Robin R Gutell

**Affiliations:** 1 Institute for Cellular and Molecular Biology, and the Center for Computational Biology and Bioinformatics, The University of Texas at Austin, Austin, TX 78712, USA; 2 Texas Advanced Computing Center, The University of Texas at Austin, Austin, TX 78712, USA; 3 Department of Computer Sciences, The University of Texas at Austin, Austin, TX 78712, USA; 4 Microsoft Corporation, 1 Microsoft Way, Redmond, WA 98052, USA

## Abstract

**Background:**

The analysis of RNA sequences, once a small niche field for a small collection of scientists whose primary emphasis was the structure and function of a few RNA molecules, has grown most significantly with the realizations that 1) RNA is implicated in many more functions within the cell, and 2) the analysis of ribosomal RNA sequences is revealing more about the microbial ecology within all biological and environmental systems. The accurate and rapid alignment of these RNA sequences is essential to decipher the maximum amount of information from this data.

**Methods:**

Two computer systems that utilize the Gutell lab's RNA Comparative Analysis Database (rCAD) were developed to align sequences to an existing template alignment available at the Gutell lab's Comparative RNA Web (CRW) Site. Multiple dimensions of cross-indexed information are contained within the relational database - rCAD, including sequence alignments, the NCBI phylogenetic tree, and comparative secondary structure information for each aligned sequence. The first program, CRWAlign-1 creates a phylogenetic-based sequence profile for each column in the alignment. The second program, CRWAlign-2 creates a profile based on phylogenetic, secondary structure, and sequence information. Both programs utilize their profiles to align new sequences into the template alignment.

**Results:**

The accuracies of the two CRWAlign programs were compared with the best template-based rRNA alignment programs and the best *de-novo *alignment programs. We have compared our programs with a total of eight alternative alignment methods on different sets of 16S rRNA alignments with sequence percent identities ranging from 50% to 100%. Both CRWAlign programs were superior to these other programs in accuracy and speed.

**Conclusions:**

Both CRWAlign programs can be used to align the very extensive amount of RNA sequencing that is generated due to the rapid next-generation sequencing technology. This latter technology is augmenting the new paradigm that RNA is intimately implicated in a significant number of functions within the cell. In addition, the use of bacterial 16S rRNA sequencing in the identification of the microbiome in many different environmental systems creates a need for rapid and highly accurate alignment of bacterial 16S rRNA sequences.

## Background

The first three RNAs identified - messenger RNA (mRNA), ribosomal RNA (rRNA) and transfer RNA (tRNA) were associated with the translation of RNA to proteins - protein synthesis. While DNA is known to carry genetic information from one generation to the next, and proteins are capable of forming three-dimensional structures that catalyze chemical reactions, the function of RNA was perceived as the carrier of the information to code for amino acids in a protein, serve as a scaffold for proteins in the ribosome, and attach amino acids to the growing protein chain during protein synthesis. All three of these RNA functions were considered to be labile and passive. However this simple perspective of RNA has been undergoing a major transformation. RNA is capable of forming complex three-dimensional structure like proteins. And like proteins these higher-order structures catalyze reactions. Now hundreds, if not thousands, of different RNA families are being identified and characterized. And RNA is being associated with many previously known functions in the cell, and equally if not more significant, RNA is being associated with many newly identified functions. This major paradigm shift in molecular and cellular biology is dramatically changing our appreciation of the machinery, mechanisms, and regulation within cells, and providing a better understanding for the normal and aberrant physiological conditions in biological organisms [1-4].

The identification and characterization of macromolecular sequences and their higher-order structures have utilized a very important principle in molecular and evolutionary biology. Different primary structures (or sequences) can form the same higher-order structure for different RNAs and proteins [5]. This comparative method is now widely used in the study for many RNAs, and is the cornerstone for the computational analysis of the deluge of nucleic acid sequences that are determined with next-generation sequencing methodology. And fundamental for many of these comparative studies is an accurate alignment of the RNA sequences that juxtapose similar structural and/or functional elements into the same set of columns.

The analysis of these alignments are used to discover patterns of structural variation and conservation, secondary and higher-order structure, phylogenetic relationships and associate function to the RNA's structure. A few of the significant discoveries made from this type of analysis include: the determination of the phylogenetic relationships for organisms that encompass all branches of the tree of life and the identification of the third domain of life - the Archaea [6], the accurate prediction of RNA secondary structures that are present within a collection of RNA sequences from the same family [7,8], the identification of new structural elements [5], the creation of pseudo-energies for many RNA structural elements and their utility in improving the accuracy of folding an RNA sequence into its secondary structure [9], and the identification of the Microbiome - the collection of microbes in different ecological environments, using 16S rRNA [10-13].

The generation of the most accurate sequence alignments is essential for the optimal analysis and interpretation of the topics listed above. The two most widely used alignment strategies are: *De novo *alignment, and template-based alignment. *De novo *sequence alignment programs (e.g. SATe [14], MAFFT [15], and Clustal [16,17]) generate multiple sequence alignments without the guide of using any preexisting alignment (template alignment).

Template-based sequence alignment programs utilize an alignment that is usually manually curated to optimize its accurate juxtaposition of nucleotides. This alignment serves as the reference to facilitate the alignment of new sequences. Several automated template-based sequence alignment services for different RNAs are available on the web: Silva [18] (16S and 23S rRNA), Greengenes [19] (16S rRNA), and RDP [20] (16S rRNA) Silva uses SINA aligner (SILVA Incremental Aligner) which is implemented with a variant of the Needleman-Wunsch algorithm [21]. Greengenes aligns a new sequence with the Nearest Alignment Space Termination [22] (NAST) algorithm. BLAST [23] is used to identify the most closely matching seed sequence and, generate a pairwise alignment. The RDP system uses Infernal [24] to align sequences based on a conserved secondary-structure.

Other research groups developed the stand-alone template-based alignment programs available for download: Infernal, ssu-align [25], and HMMER [25-27] Both Infernal and ssu-align creates consensus RNA secondary structure profiles for seed alignment, and use them to align new sequences. The program ssu-align implements additional profile hidden Markov models (profile HMMs) on the secondary structure profiles. HMMER searches for significant sequence identity with profile HMMs without using the consensus secondary structure. Infernal and HMMER are capable of building profiles and aligning any type of RNA that has an RNA alignment, while ssu-align currently is developed only for small subunit ribosomal RNA.

A variation on this approach uses a seed alignment with the correct secondary structure for that RNA family to generate a descriptor that defines the primary and secondary structure constraints. Sequences that satisfy the conditions of this descriptor are identified. Several programs that use this procedure have been developed. RNAMOT [28,29], one of the first in this family of programs, had a simple descriptor language that was easier to manually generate but was limited by the details in the structural constraints that can be identified. RNAMotif [30] and Locomotif [31] are newer programs that have a richer descriptor language with greater specificity and complexity of the RNA constraints that can be identified and distinguished. While these programs are an improvement over RNAMOT, they don't perform all of the functions needed here. They are unable to describe and identify larger RNA molecules (*e.g*. 16S rRNAs). In particular, while the program RNAMotif can search a structural model with a maximum of 100 structural elements, 16S rRNA contains over 400 structural elements. Second, these programs are also unable to encapsulate all possible variations in the RNA molecule and search candidates with acceptable performance. And third, these programs require structural descriptor to be generated manually, a process that requires a substantial amount of effort for RNA. While some of these programs have some provisions to align sequences based on similar primary and secondary structure characteristics, their performance are not adequate for aligning large numbers of large RNA sequences with great specificity.

When sequences have minimal pairwise identity with one another, *de novo *alignment algorithms have difficulty placing the nucleotides sharing common structural/functional features within each sequence into the correct columns of the alignment. In contrast, the previously determined seed alignment used in the template-based alignment algorithms has been refined to maximize the correct juxtaposition of structural, functional, and evolutionary relationships of the sequences. Thus the template-based alignment algorithms will be more accurate in the generation of new multiple sequence alignments.

Here we present two template-based alignment programs: CRWAlign-1 [32] and CRWAlign-2. As shown below, both achieve very high accuracy, even for collections of sequences that have a lower extent of sequence identity with one another. Both programs utilize the Gutell lab's RNA Comparative Analysis Database (rCAD) relational database management system that cross-indexes multiple dimensions of information, including sequence alignments, comparative secondary structures, and phylogenetic relationships [33]. CRWAlign-1 generates sequence composition profiles for each column in the alignment of different phylogenetic groups. CRWAlign-2 creates a more sophisticated profile or descriptor that contains sequence composition and secondary structure information for specific and generalized phylogenetic groups.

The CRWAlign-2 program automatically generates the structural profiles containing sophisticated constraints, searches for and creates structural models satisfying this profile, and aligns the sequences against the template. The structural information used in CRWAlign-2 is obtained from rCAD and the CRW Site (http://www.rna.icmb.utexas.edu/) [34]. The phylogenetic information in rCAD is obtained from the NCBI Taxonomy database (http://www.ncbi.nlm.nih.gov/-Taxonomy/taxonomyhome.html/). Both CRWAlign-1 and CRWAlign-2 can be used to align any type of RNA molecule that has an existing high quality alignment and for CRWAlign-2 has an accurate secondary structure model. The size of the template alignment is limited only by the number of columns and phylogenetic groups in the alignment and the size of available memory. The Gutell lab used CRWAlign-1 and CRWAlign-2 on a 16S rRNA bacteria alignment that is 10,000 columns wide and contains more than 100,000 sequences.

Our primary objective is to measure the performance of CRWAlign-1 and CRWAlign-2, and compare these performances with the most widely-used template-based alignment programs including three web-based alignment systems (Silva, GreenGenes, and RDP) and three stand-alone alignment programs (Infernal, ssu-align, and HMMER). Our analysis also included two *de novo *alignment programs (SATe and MAFFT) in our comparison. For a rigorous assessment across all alignment programs, the bacterial 16S rRNA is selected as the test set, which have at least 1,400 nucleotides per sequence in length. The results reveal that both CRWAlign-1 and CRWAlign-2 align new sequences more accurately than these other methods.

## Methods

### CRWAlign-1

CRWAlign-1 consists of two steps: 1) generate the alignment statistics of the template alignment sequences for each taxonomic group; and 2) align new sequences iteratively guided by the generated alignment statistics.

#### Alignment statistics generation

**Figure 1:**
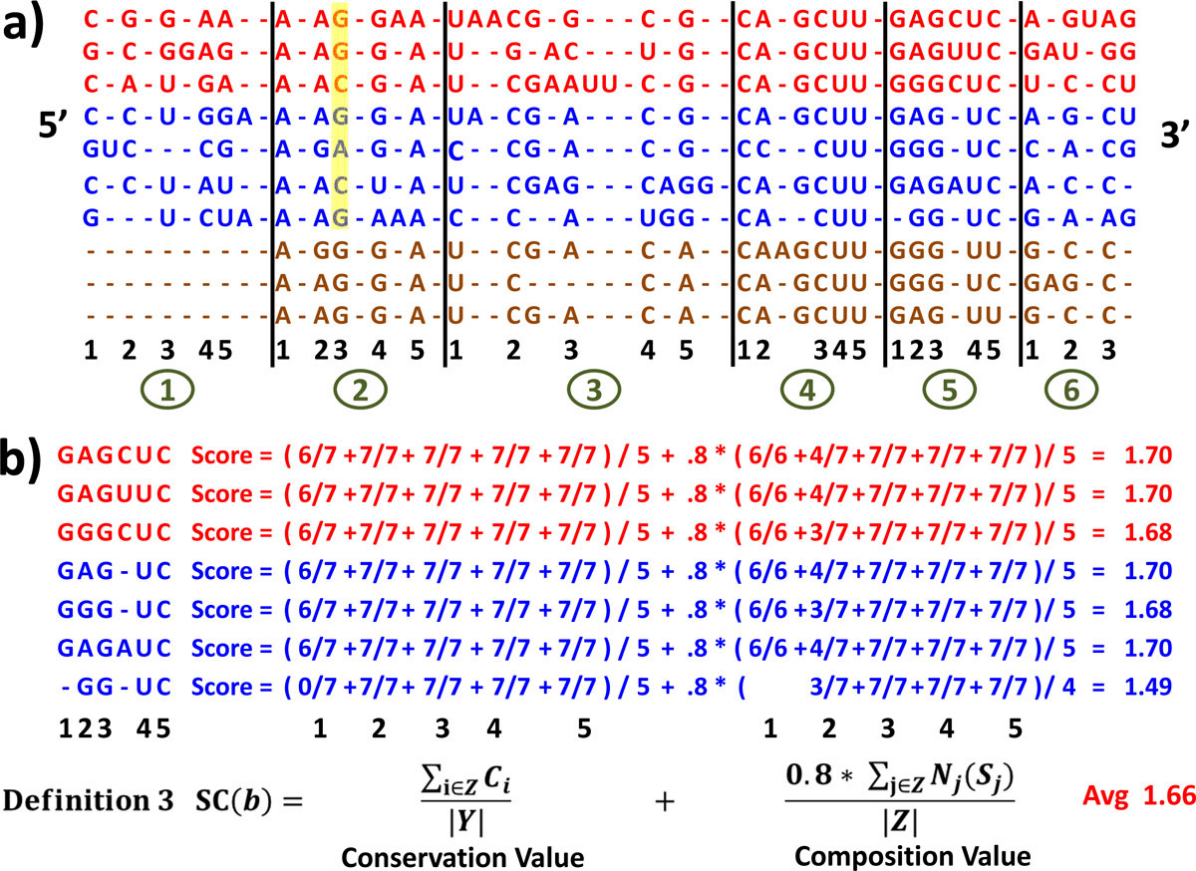
**For CRWAlign-1 - An example of the generation of alignment statistics**. a) A template alignment containing 10 sequences are used to determine conserved columns (bacillales - red, additional bacilli - blue, additional bacteria sequences - brown). The highly conserved columns are identified with a number from 1-5 immediately below the sequence alignment. Each block number is circled at the bottom of 1a. This alignment has six blocks. The first five each contain five highly conserved columns that are identified with numbers; the sixth block has three conserved columns. The yellow highlight in block 2, column 3 is described in the Methods section for definition 2; b) Calculation of the average score SC(b) [Def. 3] for block 5 in the bacilli node (see Methods section for details).

The alignment statistics are generated from the template alignment. An example of the process of generating these statistics for a template alignment consisting of 10 sequences is shown in Figure 1. The template alignment consists of sequences from three taxonomic groups under the root taxon: three bacillales sequences (red), four additional bacilli sequences (blue), and three other bacterial sequences (brown). All taxons containing the minimum number of sequences (usually 3-10) from the template alignment, and their parent taxons will be included in the statistics generation. The statistics will be generated for the following taxons: bacillales (three sequences), bacilli (parent of bacillales, seven sequences), and bacteria (root taxon, 10 sequences). The statistics generation of each taxon includes several metrics including the minimum and maximum number of nucleotides in a block, the column, conservation values (Def. 1), nucleotide composition of each column (Def. 2), and a score that is composed of the conservation and composition scores for each sequence followed by an average score for all sequences for each block (Def. 3). For each taxon, the complexity of this calculation is O(bns), where b is the length of a block, n is the number of blocks, and s is the number of sequences in the taxon.

**Definition 1: **For column *i*, the conservation value (Ci) is the number of sequences with a nucleotide in this column divided by the total number of sequences in the taxonomic node. The partial sequence starting after or ending before column *i *is excluded from the calculation.

The conservation value for each column of the template alignment is first calculated for the root taxonomic node (*i.e*. the entire alignment). Columns with conservation values greater than 80% are considered highly-conserved. The alignment is then divided into blocks that each contains the same number of highly conserved columns. The last block could have fewer columns that are highly-conserved (Figure 1a). While the sequences 8, 9 and 10 in Figure 1a are missing nucleotides at their extreme 5' end, the conservation values are not affected for these columns without nucleotides. While the number of conserved columns for each block is five for the example in Figure 1, the actual default number to obtain the highest accuracies was determined to be between 15 and 20. Statistics for the equivalent set of columns within each taxonomic node are determined although the pattern of conservation could vary between these nodes. An example of variation between nodes is shown for block 5 (Figure 1a and 1b). The red bacillales have six conserved columns while the blue bacilli and brown additional bacteria have five conserved columns.

**Definition 2: **The nucleotide composition value is the occurrence of each nucleotide (A, C, G, U) for each column, measured as the ratio of the number of sequences with each nucleotide divided by the total number of sequences with a nucleotide. This value is calculated with the equation:

(1) $${\text{N}}_{\text{i}}\left(\text{p}\right)=\frac{{\text{SC}}_{\text{p}}\left(\text{i}\right)}{\underset{\text{q}\in \left\{\text{A},\text{C},\text{G},\text{U}\right\}}{\sum }\text{S}{\text{C}}_{\text{q}}\left(\text{i}\right)},$$

where *N_ip _*is the nucleotide composition value for the *i*th column, *p*∈{A, C, G, U}, and SC_p_(i) is the number of sequences with nucleotide p in column *i*. An example for the yellow highlighted nucleotides in the conserved column 3, block 2 in Figure 1a are - {A-0%, C-33%, G-67%, U-0%} for the bacillales node (red), and {A-14%, C-29%, G-57% and U-0%} for the bacilli node (red and blue).

In addition to the conservation value (a measure of the percentage of the sequences that contain a nucleotide at a column) and the composition value (a measure of the frequency for each nucleotide type at a column), a metric is required that captures both of these measures - the conservation and nucleotide composition values for the columns within each block (Def. 3). An average is computed for the set of scores determined for each sequence within each block.

**Figure 2:**
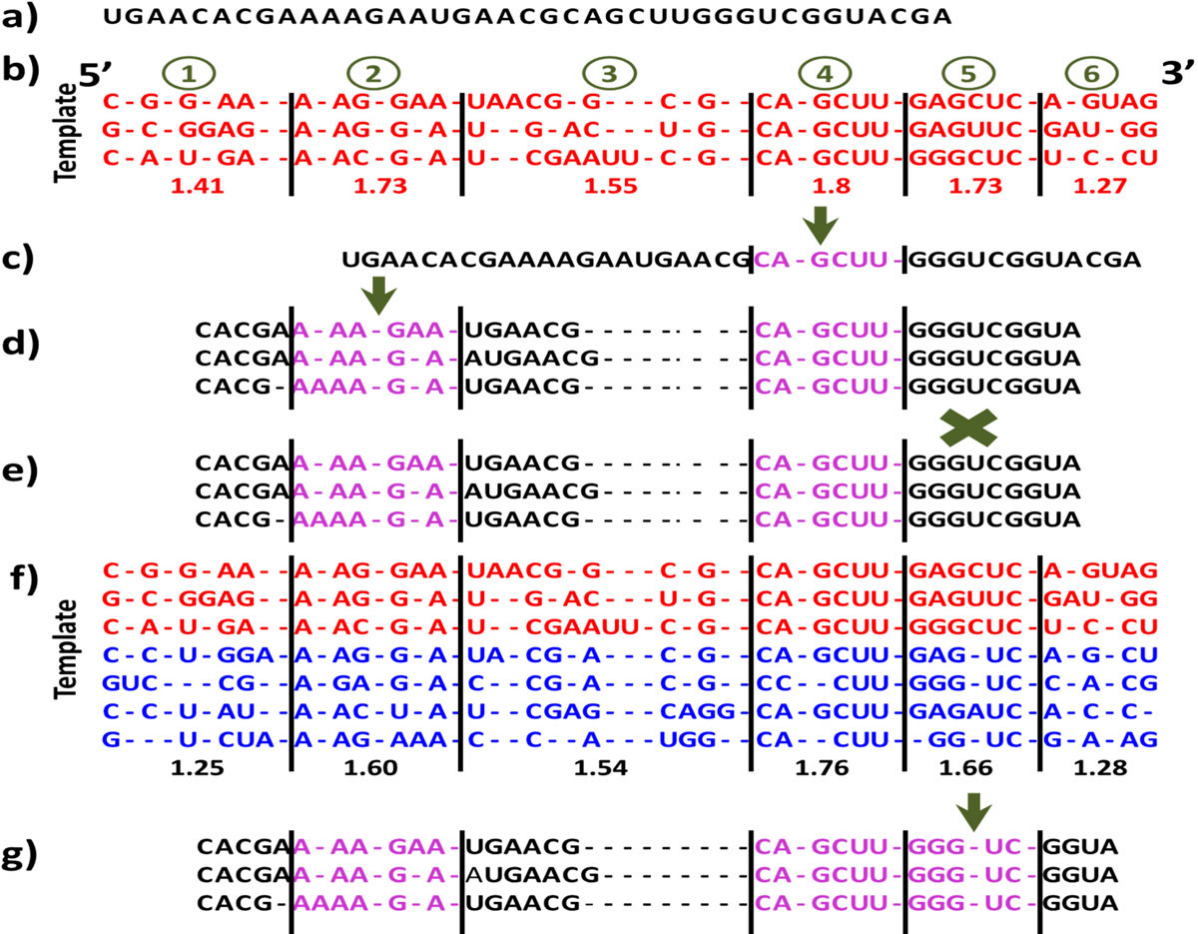
**An example of the different stages of the alignment process within CRWAlign-1**. a) a single unaligned RNA sequence; b) three aligned sequences in the template alignment at the bacillales node and the average scores for each of the six blocks; c) query sequence has a unique match for block 4 that is aligned against the template; d) matches for blocks 2 and 4 are identified; e) no matches for block 5; f) seven sequences in the template alignment at the bacilli node (three red - bacillales, four blue - other bacilli), and the average block scores; g) block 5 has a unique match (see Methods section for details).

**Definition 3: **For each block in the specified taxonomic node and subsequence S, Y is the number of conserved columns, and Z is the number of non-gap conserved columns. The equation for SC(b) - the score for each subsequence within block b is:

(2) $$\text{SC}\left(\text{b}\right)=\frac{\underset{\text{i}\in \text{Z}}{\sum }{\text{C}}_{\text{i}}}{\left|\text{Y}\right|}+\frac{0\text{.}8*\underset{\text{j}\in \text{Z}}{\sum }{\text{N}}_{\text{j}}\left({\text{S}}_{\text{j}}\right)}{\left|\text{Z}\right|},$$

where the conservation value C_i _is the percentage of sequences with a nucleotide at the *i*th column in subsequence S (see Definition 1), and N_j_(S_j_) is the nucleotide composition value at non-gapped conserved column *j *in subsequence S (see Definition 2). The value 0.8 in the second component is the weight that was determined heuristically. The SC(b) values for different blocks in Figure 2b and 2f varied from 1.25 to 1.80. A value of 1.8 is the highest possible.

Figure 1 illustrates an example of taxonomic groups within the sequence alignment, the blocks (Figure 1a - six blocks, numbered 1 - 6, block number in circle) with a fixed number of highly conserved columns (Figure 1a - highly conserved columns are numbered for each block 1 - 5), and the determination of the SC(b) values (definition 3) for block 5 for the bacillales (red) and other bacilli (blue) (Figure 1b). A few exemplary parts of the SC(b) calculation for block 5 in this example are:

• For the first - conservation value component of the equation:

○ The conservation value for column 1 is 0.86 (6/7) since six of the seven sequences have a nucleotide in this column.

○ The conservation value for columns 2, 3, 4, and 5 is 1.0 (7/7) since all seven of the sequences have a nucleotide in these columns.

○ The Y value is the set of highly conserved columns defined operationally as the columns that have a nucleotide in more than 80% of their sequences within a block. As noted earlier a block is usually created with 15 - 20 highly conserved columns. For the examples in Figures 1 and 2, blocks have five highly conserved columns.

• For the second composition component of the equation:

○ The composition value for the individual sequences in Figure 1b is 1.0 (6/6 or 7/7) for columns 1, 3, 4, and 5 for each of the sequences since only one nucleotide type is present in each of the sequences that have a nucleotide at that position.

○ In contrast, column 2 has four A's and three G's. Those sequences with an A at column 2 have 4/7 in the second column of numbers (for the second component) in Figure 1b while the sequences with a G at column 2 have a 3/7 in the second column of numbers.

○ The Z value - non-gapped conservation values is five for the first six sequences, and four for the seventh sequence.

#### Alignment algorithm

The procedure to align sequences with information from a template alignment utilizes these scores that are determined for each block and for each specific phylogenetic/taxonomic node and its parent's nodes. A description of the general strategy mimics the iterative procedure used by the Gutell lab to manually align rRNA sequences, starting in the early 1980s [7,35]. This increases the accuracy while minimizes the complexity of the alignment procedure and the compute time required to execute. An example of the alignment procedure is shown in Figure 2.

**1. Determine the phylogenetic group that the query sequence is most likely a member of**.

a. **Phylogenetic information is known: **The first step selects the most closely related phylogenetic/taxonomic group when an exact match occurs between a genus name in the template alignment and the sequence to be aligned The NCBI taxonomic information within rCAD is utilized. CRWAlign-1 selects the lowest taxon with existing alignment statistics.

b. **Phylogenetic information is not known: **If no phylogenetic information is known, the CRWAlign-1 will align the new sequence superficially against each taxon at a predefined leaf level, which is six in our experiments. The node that aligns the most nucleotides in the first stage will be used for the remainder of the alignment process for the sequence. Since the sequence to be aligned (Figure 2a, black letters indicate nucleotides that have not been aligned) is within the bacillales taxonomic group, the three aligned bacillales sequences in the initial alignment is selected to be the template (Figure 2b). This example uses the same template sequence information in Figure 1.

**2. Align sequences against the most conserved blocks at the lowest taxonomic nodes (e.g. bacillales): ** Divide and conquer approach, align the block with the highest SC(b) score for specific taxonomic nodes, then sequentially select blocks with lower scores.

a. With the lowest taxon selected (or predetermined), CRWAlign-1 attempts to align the most highly-conserved blocks with a high average score (*i.e*. the block having an average score higher than 90% of the highest allowed: 1.8 * 0.9 = 1.62). To insure the greatest accuracy at this stage, no deletion of conserved columns is allowed, and only a very limited number of insertions between conserved columns are acceptable. Aligning the most highly-conserved blocks with stringent criteria at the early stage assures that the aligned block has a high probability of being correct, reduces the complexity of the problem, and increases the speed.

b. The example shown in Figure 2b has three highly-conserved blocks in the bacillales alignment, with average scores exceeding 1.62 (blocks 2, 4, and 5). Block 4 is selected first, since its score of 1.8 is the highest. The program searches for the subsequence CAGCUU or an extremely similar sequence with a score higher than 1.62. The identified subsequence is aligned with those matching nucleotides in the template (Figure 2c, purple letters indicate nucleotides that have been aligned with CRWAlign-1). Block 2 with a score of 1.73 is aligned next (Figure 2d). Since both block 2 and 3 are variable in length, CRWAlign-1 identifies three different subsequences with acceptable sequence identities (Figure 2d).

c. The last highly-conserved block aligned in the example is block 5 (score 1.73), which is 3' of the previously aligned block 4. The 5' end of block 5 - GGGUC (Figure 2e) does not match the template sequences (G[A/G]GCUC). Since a deletion that is present in all of the sequences to be aligned is not allowed at this stage, block 5 might be aligned at the next stage.

3. Align sequences against highly conserved blocks in the parent node:

a. Once the alignment against the most conserved blocks at the lowest taxon (i.e. bacillales) is complete, the CRWAlign-1 program will attempt to align sequences to the highly conserved blocks obtained with a set of sequences in the parent taxon (i.e. bacilli). Figure 2f reveals only two blocks for the bacilli node with values above 1.62 - block 4 (score 1.76) and block 5 (score 1.66). Since block 4 has been aligned at the lower bacillales taxon, CRWAlign-1 will only attempt to align block 5. And since block 5 has five conserved columns at the bacilli node instead of six at the bacillales node, the subsequence GGGUC matches the block template without requiring any conserved column deletion (Figure 2g).

b. While blocks 2, 4, and 5 have been aligned (Figure 2g, purple letters), blocks 1, 3, and 6 have not (Figure 2g, black letters). This iterative process continues, attempting to identify and then align sub-sequences in the unaligned sequence against the most conserved blocks at the lowest taxonomic level, then the most conserved blocks in the parent taxonomic nodes, then less conserved blocks within the same taxonomic level. The CRWAlign-1 program will again attempt to align sequences to the highly conserved blocks obtained with a set of sequences in the parent taxon. For the example in Figures 1 and 2, this would be the root taxonomic node (Bacteria). The CRWAlign-1 program will attempt to align sequences against blocks in the Bacteria node that have scores above 1.62. The CRWAlign-1 program continues to the next iteration with a relaxation of the alignment criteria. The minimum block score reduced by 20% (1.8 * 0.9 * 0.8 = 1.296) and the allowance of deletions of a conserved column in a potential alignment. While the example in Figure 2 does not reveal the alignment of blocks 1, 3, and 6, it would utilize the same scoring and alignment strategy shown in Figures 1 and 2, only with relaxed criteria. This process, as noted earlier mimics the process that has been used to successfully manually align the rRNA sequences, starting in the early 1980s [7,35].

### CRWAlign-2

The CRWAlign-2 system is an enhanced and expanded version of the RNAMotif program [30] that was developed primarily to identify sequences that satisfy the secondary structure constraints in the descriptor. Our new version of this program 1) automatically generates a descriptor, 2) has a richer descriptor syntax that provides for greater specificity, 3) is capable of operating on larger RNA molecules, 4) creates secondary structure information for each sequence, 5) automatically aligns sequences based on analogous structural elements, and 6) is written in Microsoft C#. The process consists of three stages: 1) computer generate a secondary structural descriptor from an existing sequence alignment and a secondary structure model; 2) use this descriptor to identify secondary structural elements in new sequences and create secondary structure models for each sequence; and 3) use newly created secondary structure model to align sequences based on similar primary and secondary structural elements.

#### Stage 1: Computer generated secondary structure descriptor

The structural profile contains information describing various constraints applied to the canonical, regular, or standard RNA secondary structure and relevant taxonomy. The syntax of the profile is based on the original RNAMotif program [30] but enhanced significantly. The most important enhancements include: a) the structural constraints (*e.g*. the length of helix/unpaired region, the mispair number for helix) are described more accurately and assigned with a weight score indicating its frequency; b) a weight score cutoff section defines the lowest score of an elongating structural model (to significantly reduce the running time of structure identification process); and c) a phylogeny section describes the most relevant constraints applying on various phylogenetic groups.

**Figure 3:**
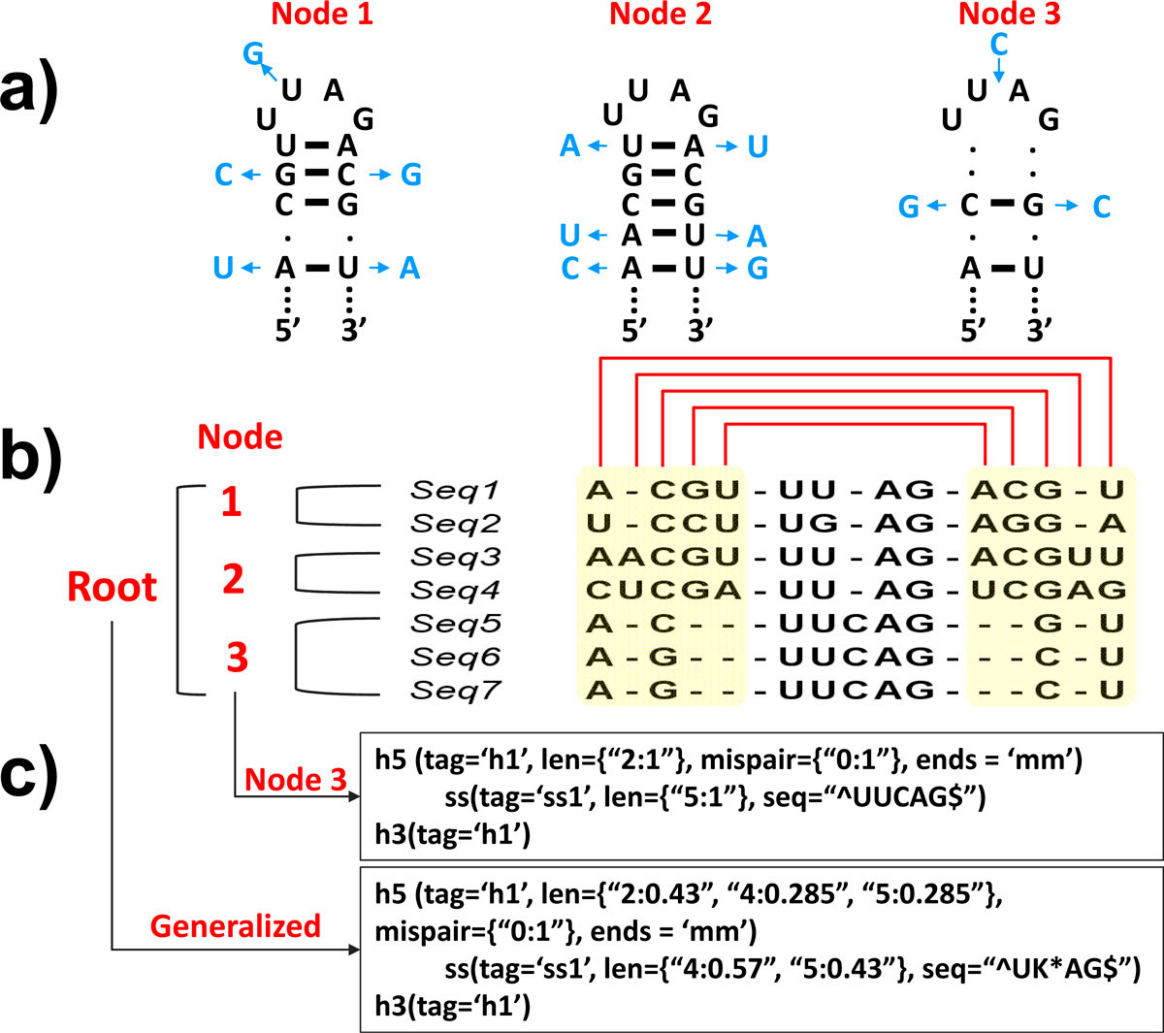
**For CRWAlign-2, (a) secondary structure diagrams for each of the three phylogenetic nodes**. ***Seq1 ***in node 1, ***Seq3 ***in node 3, and ***Seq5 ***in node 3 are shown with black nucleotides; Blue nucleotides reveal the differences between the first sequence in each node (Seq1, Seq3, and Seq5) and the other sequences within each node; (b) Template sequence alignment with seven sequences distributed over three phylogenetic nodes. Red lines above the alignment indicate columns in alignment that form a base pair; (c) RNAMotif structural descriptors for node 3 and for all seven sequences (root).

Figure 3 reveals the relationship between structural elements that occur within the same region of an RNA molecule and the template sequence alignment. Aligned sequences in the same phylogenetic nodes are grouped together, *i.e*. seq1-2 under node 1, seq3-4 under node 2, and seq5-7 under node 3. For each of the three nodes, variation in nucleotide composition is shown in the secondary structure diagram and in the alignment. The first sequence in each node is shown in black, with variations within each node shown in blue (Figure 3a and 3b). At the onset, a generalized structure descriptor (Figure 3c) that contains the constraints, such as the length of helix and unpaired region, mispair number, nucleotide conservation, *etc*, for all sequences in the template alignment is created. The structural descriptors for all phylogenetic nodes (Nodes 1, 2, 3 in Figure 3c) are created to provide the specific structural constraints for each of the phylogenetic nodes.

#### Stage 2: Identify secondary structural elements and create secondary structure models

**Figure 4:**
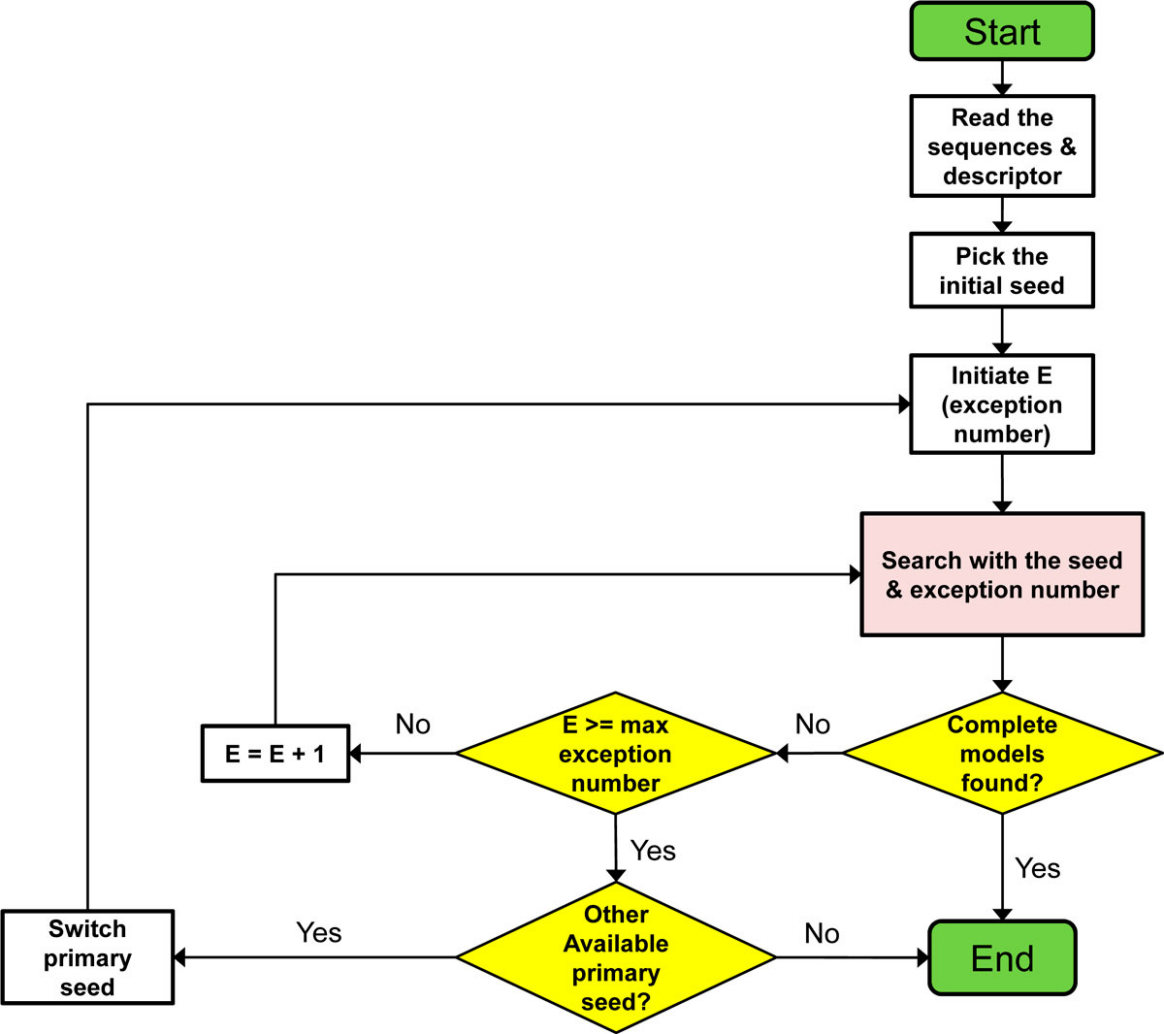
**The flowchart of the complete structural model identification process for CRWAlign-2**. The program reads the structural descriptor and sequences to be aligned, prioritizes structural elements in the descriptor to build seed points, and iteratively searches for complete structural models on the sequences that satisfy all structural constraints defined in the descriptor (see text in Methods section for details).

The program attempts to identify the structural elements within the descriptor in a sequence. As shown in Figure 4, the first step is to read the sequence (*e.g*. GenBank entries) and the descriptor that was generated in Stage 1.

The next step prioritizes or ranks the structural elements in the descriptor by their stringency, as measured with a probability score for each structural element. The more stringent, the less likely it will occur by chance, and thus has a lower probability score. The identification of the structural model starts with the structural elements with the lowest probability scores, which serve as the initial seed or nucleation points. The program then attempts to identify the structural elements that are adjacent to these initial seeds. A structural element can be either a base paired region (*e.g*. a helix) or an unpaired region (*e.g*. a hairpin, internal, or multistem loop). When the attempted extension is successful, then this larger extended structure becomes the seed for the next round. This extension of the structural model iterates either to a complete structural model, as defined by the identification of all of the structural elements in the descriptor. Or when the extension on the 5' and/or 3' ends of the seeds cannot be extended, and then the process is terminated, resulting in a partial structure model.

Consistency checks, an evaluation of the fitness of the constraints for each structural element with the identified structural model, are performed at each round of extension. During the extension, it is possible that a conflict occurs where a putative structural element identified in say round 5 conflicts with structural elements identified in round 7. The entire structure model is terminated resulting in a partial solution.

Abnormal or aberrant insertions/deletions or nucleotide composition can occur, stopping the continuous elongation. While the original RNAMotif would abort, and not report a partial or complete structure model, our newer program allows the user to permit exceptions to the descriptor. The number of exceptions, variances from the canonical or regular structure model can be defined by the user. As expected, the number of complete or partial structure models will increase as the number of allowed exceptions is increased. Thus while this number should be minimal, it is extremely useful to have this option to permit the identification of structural models that are truly exceptions to the norm.

The specificity and resolving power for this identification is greatest when the phylogenetic information is known, since the descriptor has more structural constraints (see Figure 4). In contrast, the generalized descriptor identifies more sequences and requires more computational time.

It is important to note that this program not only identifies sequences that contain the structure model in the descriptor, but also creates a structure model for those sequences that have one. This feature thus allows a very large number of comparative structure models to be generated. These comparative structure models are used to evaluate the accuracy of RNA folding algorithms [9,36].

#### Stage 3: Aligning sequences based on similar primary and secondary structural elements

As noted earlier, our version of the enhanced RNAMotif program is capable of aligning sequences based on a common secondary structure. With the complete structural models determined in stage 2, the sequence to be aligned is split into multiple fragments. Each fragment represents a specific structural element in the secondary structure. After the fragments are generated, this alignment program identifies the previously aligned template sequence that is most similar to the sequence to be aligned, based on the length of the fragment and the sequence conservation. The alignment of the new sequence against the template will first attempt to juxtapose the pairing regions (*e.g*. helices), regardless of sequence conservation. In contrast, sequence conservation in the unpaired regions is the primary factor in the juxtaposition of sequences.

## Results

The accuracy of the alignment results, the running time of the program executions and the scalability for large datasets of both CRWAlign-1 and CRWAlign-2 has been evaluated. The result has been compared to eight existing widely-used alignment programs.

### Programs compared

There are eight alignment programs included in the comparison with CRWAlign-1 and CRWAlign-2: ssu-align, infernal, HMMER, MAFFT, SATe, RDP, Silva, and GreenGenes. Six are template-based programs: ssu-align, infernal, HMMER, Silva, RDP, and GreenGenes while the other two (MAFFT and SATe) align sequences from scratch. Among the six template-based programs, ssu-align, Innfernal and HMMER are stand-alone and available for download at http://selab.janelia.org/software.html). Silva, RDP and Greengenes are only available as web-servers and able to only align 16S rRNA while the remaining seven programs (either template-based or *de novo*) are capable of aligning any type of RNA sequences. Our analysis of these programs and web sites used the default parameters.

### Calculating the accuracy of an alignment

Both test and template data sets are random subsets of the bacterial 16S rRNA alignment available at the Comparative RNA Web (CRW) Site (see Table 1 for detail). None of the sequences in the test set are present in the template set (i.e. no overlap between a test and template set). In measuring the template size effect, small template sequence alignments are always subsets of any larger template alignment (e.g the 500 16S rRNA template alignment was a subset of the 2000 16S rRNA template alignment).

**Table 1:** Sequences in template alignments and used for testing.

RNA Molecule	Template Sequences			Unaligned Test Sequences		
	
	Count	Avg Length	# of Taxonomic Leafs	Count	Avg Length	# of Taxonomic Leafs
	250	1447.3	188	500	1446.1	320
				
16S Bacterial rRNA	500	1447.4	324			
				
	1000	1449.4	593	1000	1448.1	598
				
	2000	1449.2	1154			

No overlap occurs between sequences tested and sequences in template alignments.

The accuracy of the alignments generated for this analysis is evaluated through pairwise sequence comparisons. Given a pair of sequences *i *and *j*, the pairwise sequence identity for sequences *i *and *j *is defined as:

(3) $$PSI_{ij}=\frac{\left|\text{B}\right|}{\left|\text{E}\right|}$$

Where B is the set of columns that contain a nucleotide from both sequences *i *and *j*, and E is the set of columns that contain a nucleotide from either sequence *i *or *j*. The pairwise sequence accuracy is defined as

(4) $$\text{Accuracy}=\frac{\left|\text{S}\right|}{\left|\text{E}\right|}$$

where *S *is the set of columns in sequence *i *and *j *that have the identical stack in the test alignment and the correct alignment. For example, if nucleotide 55 (G) of sequence *i *is stacked with nucleotide 63 (C) of sequence *j *in the correct alignment, then the test alignment must have nucleotide 55 (sequence *i*) only stacked with nucleotide 63 (sequence *j*).

### Accuracy comparison with other methods

**Figure 5:**
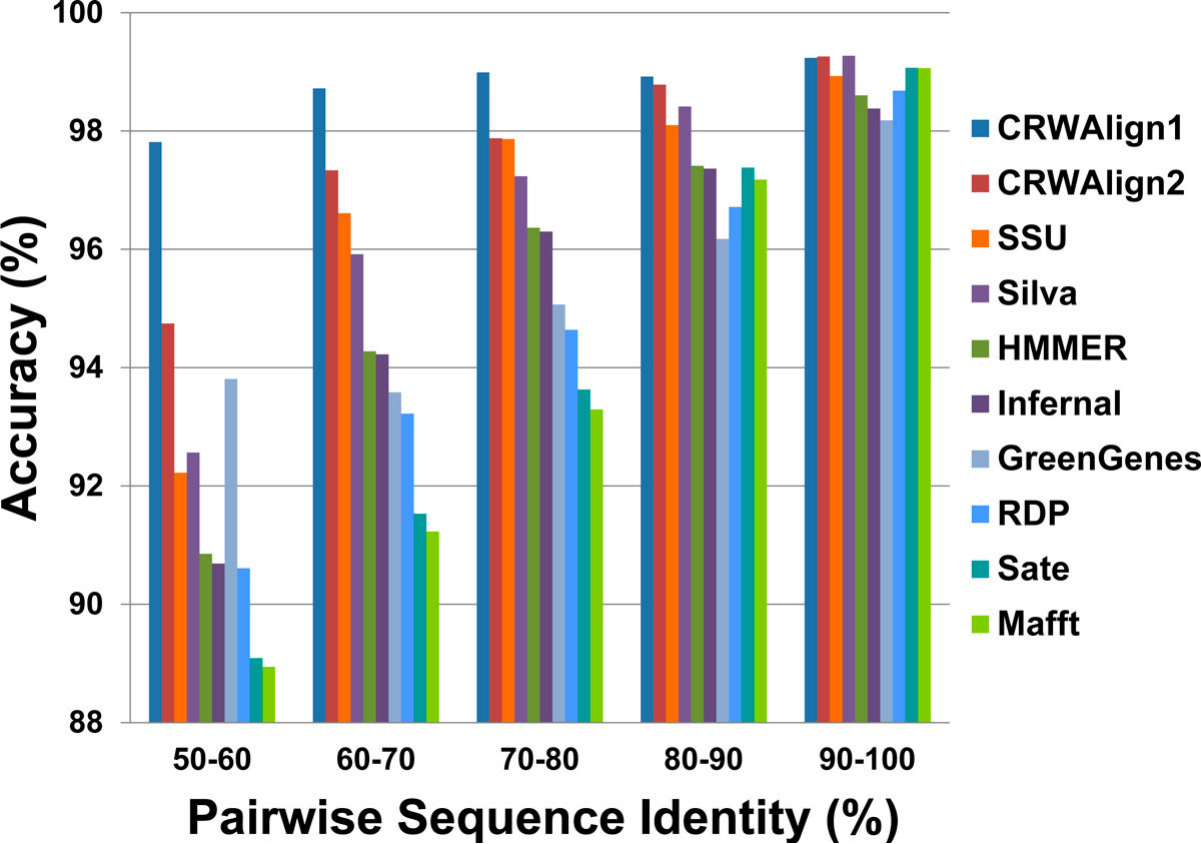
**The pairwise sequence accuracies for alignments generated with CRWAlign-1, CRWAlign-2, and eight other alignment programs**. Accuracies were evaluated for sequences with five pairwise sequence identities, 50-60%, 60-70%, 70-80%, 80-90%, and 90-100%. Alignments contain 1,000 bacterial 16S rRNA sequences.

Several alignment programs have limitations in the type of sequence to be aligned. Since the bacterial 16S rRNA is the only RNA that can be aligned by all ten programs, it is used in this study. A test set consisting of 1000 bacterial 16S rRNA sequences was aligned by each alignment program. The accuracies of the generated alignments were calculated based upon the pairwise sequence identity ranges (Figure 5). The four programs that accept template alignments (CRWAlign-1, CRWAlign-2, HMMER and Infernal) were given three template alignments with different size (250, 500, and 2000 sequences), and the best results of each are presented in Figure 5.

All 10 programs achieved high accuracy in the 90-100% pairwise sequence identity range. CRWAlign-1, CRWAlign-2 and Silva outperformed the other programs with ~99.25% accuracy, which was 0.2% (for SATe, Mafft) to 1.0% (for GreenGenes) higher than the other seven programs. In the 80-90% identity range, CRWAlign-1 (98.9%) and CRWAlign-2 (98.8%) were superior to the other eight programs, including Silva, by 0.5% (Silva) to 2.7% (GreenGenes). In the 70-80% sequence identity range, CRWAlign-1 achieved ~99% accuracy, 1% above the second best program. CRWAlign-2 and ssu-align followed with 97.9% accuracy. The other seven programs varied from 1.8% (Silva) to 5.7% (Mafft) less than the top three programs. In 60-70% and 50-60% sequence identity ranges, both CRWAlign-1 and CRWAlign-2 beat every other program in accuracy, while CRWAlign-1 achieved higher accuracy scores than CRWAlign-2. While the *de novo *programs (MAFFT, SATe) were able to align sequences as well as the template-based programs in the high sequence identity range (80-90% and 90-100%), their accuracies were remarkably lower than the template-based accuracies for sequences with low pairwise identity.

The structure-based alignment program, CRWAlign-2, identified the complete secondary structural models in the unaligned sequences and used the structural model to align the sequences. This required the unaligned sequence to be "complete": it should contain every structural element (*e.g*. helix, unpaired region) in the reference sequences. A significant percentage of the unaligned sequences are not complete. Thus it is anticipated that the CRWAlign-2 will align complete sequences with higher accuracy.

### Effect of template size on accuracy

**Figure 6:**
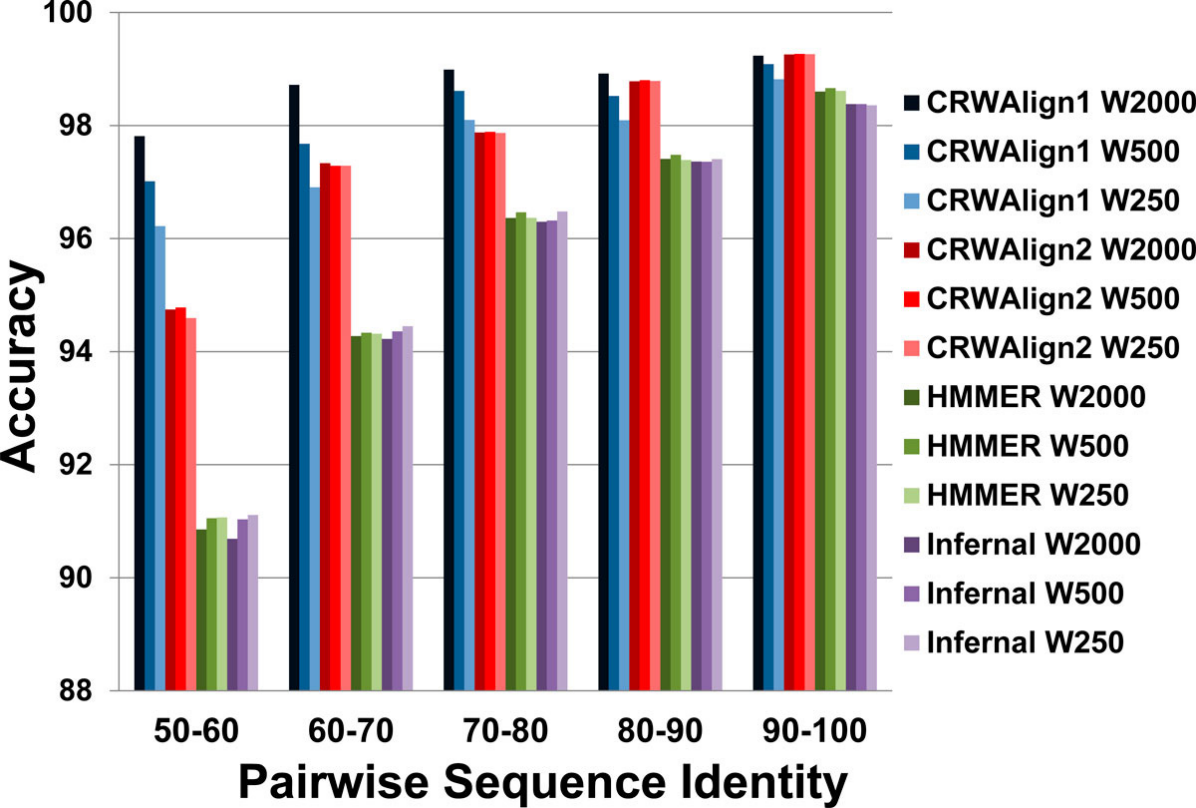
**The pairwise sequence accuracies for alignments generated with CRWAlign-1, CRWAlign-2, HMMER, and Infernal were determined**. The alignments contain 1,000 bacterial 16S rRNA sequences. Three different template sizes (250, 500, and 2,000 sequences) were evaluated for five pairwise sequence identities, 50-60%, 60-70%, 70-80%, 80-90%, and 90-100%.

To determine the influence of the template size on the accuracy for CRWAlign-1, CRWAlign-2, HMMER and Infernal, each program was analyzed to align 1000 bacterial 16S rRNA sequences with three different template alignments containing 250, 500 and 2000 bacterial 16S rRNA sequences. As shown in Figure 6, CRWAlign-2, HMMER, and infernal achieved nearly identical accuracies in all three template alignments, while CRWAlign-2 was always superior to HMMER and Infernal.

In contrast, CRWAlign-1 aligned sequences more accurately when the template alignment contained more sequences. In the 50-60% and 60-70% pairwise sequence identity ranges, the large template alignment helped CRWAlign-1 to obtain much higher accuracy. WhileCRWAlign-1 performed best with the large template alignment containing 2,000 sequences, its results with 250 sequence template alignment was still superior or as good as other alignment programs in this study (Figure 6).

### Comparisons of the run time of programs

**Figure 7:**
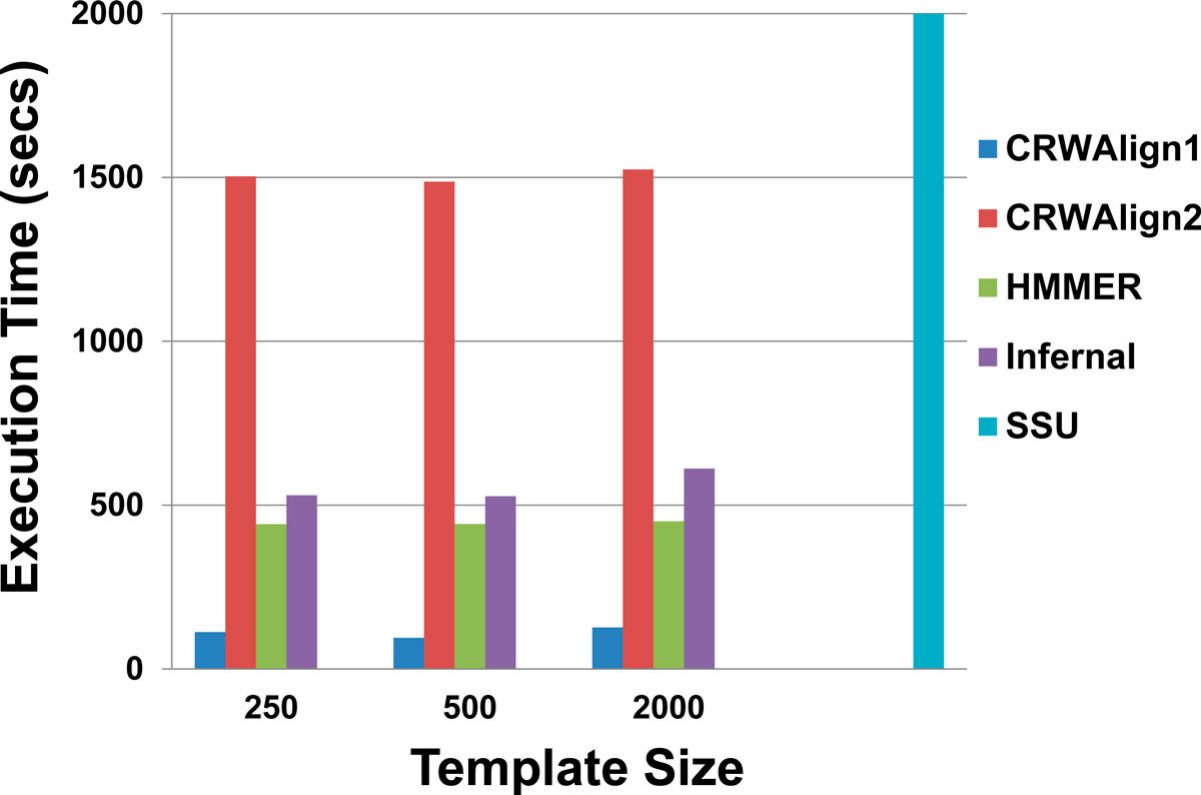
**The total execution time of aligning 1,000 bacterial 16S rRNA sequences for five alignment programs with three different template sizes (250, 500, and 2,000 sequences)**.

The execution time required to align 1000 bacterial 16S rRNA sequences were determined for CRWAlign-1, CRWAlign-2, and the three stand-alone programs, HMMER, Infernal and ssu-align. There were three data points collected for CRWAlign-1, CRWAlign-2, HMMER, and Infernal that accept user-defined template alignment, while only one data point for ssu-align since it integrated the profile (default template) into the program. Due to platform requirements and software dependencies, CRWAlign-1 and CRWAlign-2 were tested on Windows Server 2008 R2 Enterprise (64 bit) with an Intel Xeon x7550 @ 2 GHz. HMMER and Infernal were run on a Linux platform (Ubuntu 11.10, 32 bit) with an Intel Core i7 920 @2.67 GHz. The ssu-align program was run on Solaris 10.0 with an Intel Xeon processor 5400. These three server configurations have very comparable speeds. Figure 7 showed that CRWAlign-1 ran more than 15 times faster than ssu-align, and on average, four times faster than HMMER, and five times faster than Infernal. CRWAlign-2 created the complete secondary structure for each sequence to be aligned. The identification of structural models was an iterative process and required significant amount of computational time, which was still faster than ssu-align. While the comparative structural models generated with CRWAlign-2 are essential for the alignment of sequences, these structure models can be used for other applications, *e.g*. to improve and evaluate RNA folding algorithms [9].

### Scalability of CRWAlign

CRWAlign-1 consists of two phases: 1) analyzing the template alignment to generate statistics, and 2) aligning sequences. The CRWAlign-2 programconsists of three phases: 1) the generation of structural descriptor, 2) the identification of complete structural models for each sequence, and 3) aligning sequences. The total run time of both CRWAlign-1 and CRWAlign-2 is expected to be sensitive to the template size as well as the number of sequences to be aligned.

**Figure 8:**
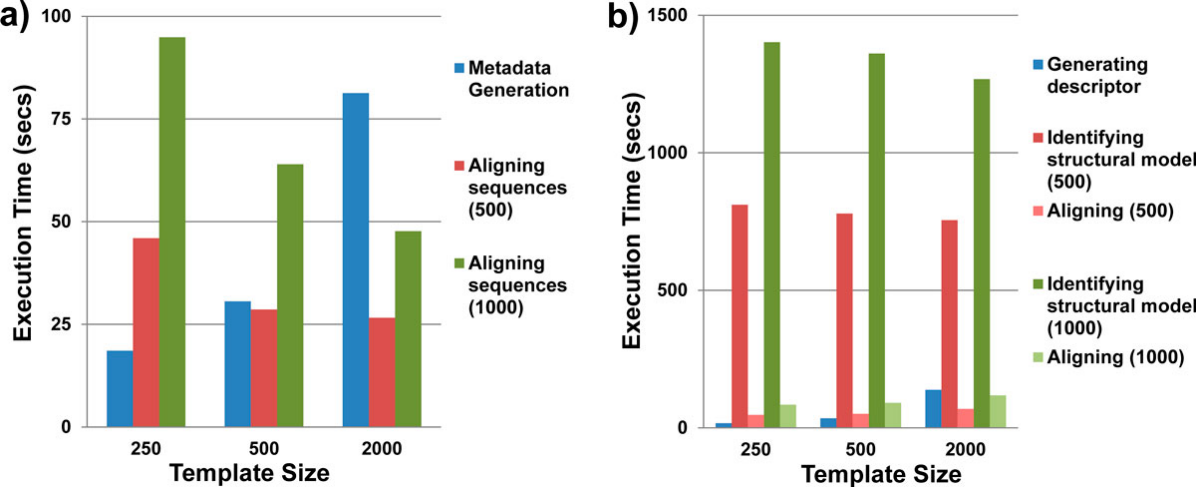
**The execution time of the different phases for a) CRWAlign-1 and b) CRWAlign-2 programs in aligning two test sets (500 and 1,000 bacterial 16S rRNA sequences) with three different template sizes (250, 500 and 2000 sequences)**.

The execution time of CRWAlign-1 and CRWAlign2 for aligning two test sets with varying size(500 and 1,000 bacterial 16S rRNA sequences) using three different template alignments (250, 500 and 2,000 sequences) were determined and shown in Figure 8. For CRWAlign-1, the computational cost of generating the statistics increased linearly with the number of sequences in the template alignment, while the execution time of the alignment phase was linear to the number of sequences to be aligned (Figure 8a). In addition, CRWAlign-1 was able to align sequences faster as the number of sequences in template alignment increased. This increase in speed was most prominent when the size of template alignment is relatively small, e.g. the template size was increased from 250 to 500. There will be a diminishing return as the size of the template alignment grows to a large number.

Similar results were observed in CRWAlign-2 (Figure 8b). The computational time of generating the structural descriptor was linear to the number of sequences in the template alignment, while the identification of structural models and aligning sequence increased computational cost linearly to the number of sequences to be aligned. The identification of structural models was the most time consuming stage.

## Discussion and conclusion

While manual curation has been used to create the optimal juxtaposition of similar structural elements in the sequence alignments available at the Comparative RNA Web (CRW) Site [34], this process can require a significant amount of time. With the development of next generation sequencing technology that generates exceedingly large amounts of sequence data, manual curation is not feasible. Thus it is an essential and challenging task to develop alignment programs that will accurately and quickly facilitate the creation of sequence alignment.

In addition to CRWAlign-1 that aligns sequences [32], we have developed a second template-based alignment program - CRWAlign-2 that utilizes primary and secondary structure constraints. Both programs significantly outperform analogous programs in accuracy. The CRWAlign-1 program utilizes multiple dimensions of data including sequence composition, column conservation and phylogenetic information. The CRWAlign-2 program utilizes secondary structural information, sequence composition and phylogenetic information. Both programs retrieve the required information from rCAD, and use it to generate the statistics and structural descriptor which helps aligning new sequences.

The accuracy of both CRWAlign-1 and CRWAlign-2 is tested on a set of 16S bacterial rRNA sequence alignments and compared with various template-based and *de novo *RNA sequence alignment programs (Figure 5). The competing programs get similar accuracy to both CRWAlign programs when the sequence identity was over 90%. However, for lower sequence identity, the other programs are less accurate than CRWAlign-1 and CRWAlign-2. Even for sequence identity ranges of 50-60%, both CRWAlign programs are >94.7% accurate.

While the CRWAlign-2 program creates complete secondary structural models for its alignment process, the secondary structure information for each sequence is valuable for the development of RNA secondary structure prediction programs [9,37-40], and for the determination of structural statistics (http://www.rna.ccbb.utexas.edu/SAE/2D/index.php). The structure information created from CRWAlign-2 can be converted into different formats, such as bpseq, alden, rnaml, ct, and bracket (http://www.rna.icmb.utexas.edu/DAT/3C/SBPI/[34]), further increasing their utility. Currently the Gutell lab's Comparative RNA Web (CRW) Site has nearly 55,000 structure files in these multiple formats. The CRWAlign-2 system has the potential to increase the number of comparative structure model files to more than 1,000,000.

The execution time of the CRWAlign-1 program is significantly less than the three programs available for download, ssu-align, HMMER, and Infernal (Figure 7). CRWAlign-2 creates the comparative structural model for each sequence to be aligned, requiring more computational cost as noted earlier. The performance of both CRWAlign programs scale linearly with the number of sequences 1) to be aligned and 2) in the template alignment (Figure 8).

Since the rate of sequence data generated with next-gen sequencing is increasing faster than Moore's law, it is essential that more accurate and fast automatic alignment methods be developed. And given that multiple-dimensions of information are available in systems like rCAD [33], we have developed two systems to address this challenge and need.

## Competing interests

Microsoft Research provided an external research grant to RRG. Although one of the authors, SO, is employed by Microsoft, no one else at Microsoft or Microsoft Research have any competing interests with any aspect of this manuscript. The other authors have declared that no competing interests exist.

## Authors' contributions

Lei Shang:

• Project design and contributed to the preparation of the manuscript

• Developed computer programs

• Analysed the data

David P. Gardner:

• Project design and contributed to the preparation of the manuscript

• Developed computer programs

• Analysed the data

Weijia Xu:

• Project design and contributed to the preparation of the manuscript

• Analysed the data

Jamie J. Cannone:

• Prepared data for project

• Consulted on program development, general comments about the study, and revised the manuscript

Daniel P. Miranker:

• Consulted on program development, general comments about the study, and revised the manuscript

Stuart Ozer:

• Consulted on program development, general comments about the study, and revised the manuscript

Robin R. Gutell:

• Project design and contributed to the preparation of the manuscript

• Analysed the data

## Declarations

The publication costs for this article were funded by a gift from Microsoft Research.

This article has been published as part of *BMC Systems Biology *Volume 7 Supplement 4, 2013: Selected articles from the IEEE International Conference on Bioinformatics and Biomedicine 2012: Systems Biology. The full contents of the supplement are available online at http://www.biomedcentral.com/bmcsystbiol/supplements/7/S4.
